# Influence of the Sevoflurane Concentration on the Occurrence of Epileptiform EEG Patterns

**DOI:** 10.1371/journal.pone.0089191

**Published:** 2014-02-26

**Authors:** Ines Kreuzer, W. Alexander Osthaus, Arthur Schultz, Barbara Schultz

**Affiliations:** Department of Anaesthesiology and Intensive Care Medicine, Hannover Medical School, Hannover, Germany; Hangzhou Normal University, China

## Abstract

**Objectives and Aim:**

This study was performed to analyse the effects of different sevoflurane concentrations on the incidence of epileptiform EEG activity during induction of anaesthesia in children in the clinical routine.

**Background:**

It was suggested in the literature to use sevoflurane concentrations lower than 8% to avoid epileptiform activity during induction of anaesthesia in children.

**Methods:**

100 children (age: 4.6±3.0 years, ASA I–III, premedication with midazolam) were anaesthetized with 8% sevoflurane for 3 min or 6% sevoflurane for 5 min in 100% O_2_ via face mask followed by 4% sevoflurane until propofol and remifentanil were given for intubation. EEGs were recorded continuously and were analysed visually with regard to epileptiform EEG patterns.

**Results:**

From start of sevoflurane until propofol/remifentanil administration, 38 patients (76%) with 8% sevoflurane had epileptiform EEG patterns compared to 26 patients (52%) with 6% (p = 0.0106). Epileptiform potentials tended to appear later in the course of the induction with 6% than with 8%. Up to an endtidal concentration of 6% sevoflurane, the number of children with epileptiform potentials was similar in both groups (p = 0.3708). The cumulative number of children with epileptiform activity increased with increasing endtidal sevoflurane concentrations. The time from start of sevoflurane until loss of consciousness was similar in patients with 8% and 6% sevoflurane (42.2±17.5 s vs. 44.9 s ±14.0 s; p = 0.4073). An EEG stage of deep anaesthesia with continuous delta waves <2.0 Hz appeared significantly earlier in the 8% than in the 6% group (64.0±22.2 s vs. 77.9±20.0 s, p = 0.0022).

**Conclusion:**

The own analysis and data from the literature show that lower endtidal concentrations of sevoflurane and shorter administration times can be used to reduce epileptiform activity during induction of sevoflurane anaesthesia in children.

## Introduction

The volatile anaesthetic sevoflurane is widely used for mask induction in paediatric anaesthesia. Advantages of sevoflurane are that it is nonpungent and that it is characterized by a lack of upper airway irritation [1]. The use of high concentrations of 7–8% sevoflurane has been recommended to accelerate the loss of consciousness and to minimize the risk of body movements, agitation, breath holding, and coughing [2], [3]. The cardiac output is better maintained at deep anaesthesia levels compared with halothane [1].

Several studies showed that one drawback of induction with sevoflurane can be the occurrence of epileptiform electroencephalogram (EEG) patterns [3], [4], [5], [6], [7], [8], [9]. Epileptiform potentials are observed under deep sevoflurane anaesthesia, sometimes accompanied by tonic-clonic movements [10], but most often without clinical signs [11]. Hyperventilation [4], speed of induction [7], high alveolar concentrations of sevoflurane [4], [8], [12], and female gender [2] are discussed in the literature as factors which may activate seizure-like motor activity as well as EEG abnormalities [13] during sevoflurane administration. As it is still not clear whether these EEG changes are of clinical relevance, some authors recommend to avoid them [14]. Holzki and Kretz [12] considered the use of sevoflurane in concentrations not higher than 5.0–5.5% in N_2_O during the induction period in order to prevent the occurrence of epileptiform EEG activity.

In an own study, inductions of anaesthesia carried out with 6% or 8% sevoflurane and without N_2_O as part of the clinical routine were to be compared with regard to epileptiform EEG phenomena. It was to be analysed, if the number of children with epileptiform potentials, the kind of epileptiform patterns and their frequency of occurrence was different. Furthermore, the times until loss of consciousness (LOC) and to the first occurrence of epileptiform patterns were to be evaluated. A detailed knowledge of the development of epileptiform activity under different sevoflurane concentrations could help to avoid these potentials.

## Materials and Methods

### Ethics Statement

Approval from the ethics committee of Hannover Medical School was obtained for this study.

### Patients and Study Design

Data of 100 American Society of Anesthesiologists (ASA) physical status I - III children undergoing elective surgery in the paediatric theatre were studied with written parental consent. The children were older than one year. Induction of anaesthesia with sevoflurane was performed according to standard procedures in the department.

All children received 0.5 mg/kg body weight midazolam (maximum dose 10 mg) as oral premedication 45 minutes before induction of anaesthesia.

Standard monitoring, including non-invasive blood pressure, electrocardiogram (ECG), pulse oximetry, and EEG, was established before induction of anaesthesia. The anaesthetic circuit was prefilled with 8% or 6% sevoflurane in 100% O_2_ for 5 minutes. The patients received mask induction with either 8% sevoflurane for 3 min or 6% sevoflurane for 5 min followed by 4% sevoflurane until propofol and remifentanil were given before the intubation. Endtidal sevoflurane concentrations were documented every minute for 5 minutes starting with the beginning of the sevoflurane administration. A fresh gas flow of 4 l/min was used. The time at which the LOC occurred was noted. Criteria of LOC were closed eyes and absence of voluntary movements. After LOC, the children breathed spontaneously or manually assisted to achieve normoventilation (endtidal CO_2_ 35–40 mmHg). A peripheral vein was cannulated and an isotonic balanced electrolyte solution with a low glucose concentration of 1% (Elektrolyt-Infusionslösung 148 mit Glucose 1 PÄD, Serumwerk Bernburg AG, Bernburg, Germany) was started. For tracheal intubation, a bolus of 3 mg/kg propofol, 1 µg/kg remifentanil, and 0.5 mg/kg atracurium was given. Anaesthesia was maintained with a constant dosage of 0.15 µg/kg/min remifentanil and with sevoflurane according to clinical requirements.

Exclusion criteria were a patient age of less than 1 year and a history of febrile convulsions or any other seizure activity.

### EEG Monitoring

The EEGs were recorded with the EEG monitor Narcotrend® (MT MonitorTechnik, Bad Bramstedt, Germany) [15]. After the skin had been prepared with alcohol and abrasive paste, two standard ECG electrodes were placed on the patient’s left and right forehead and a third electrode as reference in the middle. The impedances were below 6000 ohms.

### Visual EEG Analysis

All 100 EEGs were analysed visually by a rater (B. S.) who holds the EEG certificate from the German Society of Clinical Neurophysiology and Functional Imaging. She was unaware of the induction type and made the visual assessments retrospectively.

**Figure 1:**
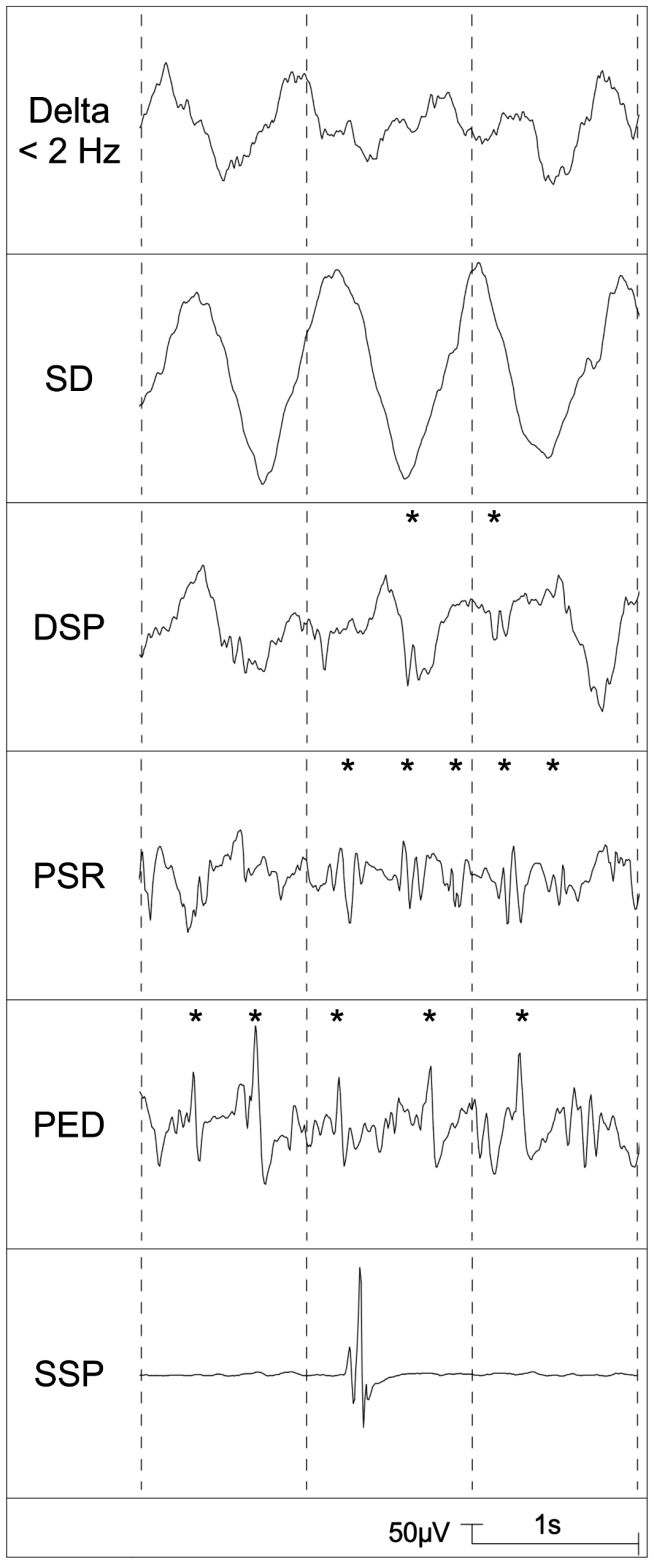
Typical examples for the EEG patterns delta <2 Hz, slow delta (SD), delta with spikes (DSP), rhythmic polyspikes (PSR), periodic epileptiform discharges (PED), and suppression with spikes (SSP).

Based on the descriptions of Vakkuri et al. [4], the following EEG patterns which were regarded as epileptiform patterns [2] were distinguished: DSP (delta with spikes; delta activity of any frequency with regular or irregular spikes), PSR (rhythmic polyspikes; waveform with more than two negative and positive deflections appearing at regular intervals, associated with slow wave or mixed frequency EEG activity between spike complexes), PED (periodic epileptiform discharges), SSP (suppression with spikes; short episodes consisting mostly of a single spike appearing during complete EEG suppression). Examples are presented in **Figure 1**. Additionally, the recordings were analysed with regard to the pattern SD (slow delta; delta activity <2.0 Hz with a regular sinusiodal pattern and high amplitudes) (**Figure 1**) [4].

For each EEG measurement, the following three sections were defined. Section A lasts from the start of the sevoflurane administration until the beginning of the propofol injection. Section B comprises the time of the propofol injection, and of following EEG suppression periods and bursts, and ends with the transition to a continuous EEG indicated by the last suppression period which has a duration of at least 2 s. The ensuing section C lasts until the end of sevoflurane administration.

For each epileptiform pattern, the time from the start of sevoflurane administration until the pattern occurred for the first time was noted. The number of seconds with each pattern was determined. Four groups were built: 0 s, 1–2 s, 3–9 s, and >9 s with epileptiform patterns.

Furthermore, it was determined at which time an EEG pattern of deep hypnosis consisting of continuous delta waves <2 Hz was reached.

The analysis focused on the induction period. In section C, the EEGs were assessed with regard to continuous periods of more than 60 s length with epileptiform activity.

### Statistical Analysis

Statistical analysis was performed using the statistical software SAS (SAS Institute, Cary, USA), version 9.3.

For nominal data, statistical analysis was performed by Fisher’s exact test. Numerical data were analysed by t-test, Wilcoxon signed rank sum test, or two-way analysis of variance (ANOVA). Statistical significance was defined as p<0.05.

Quantitative variables were expressed as mean and standard deviation or median with minimum and maximum. One of the authors (A. S., biostatistician and anaesthesiologist) was responsible for the statistical analysis.

## Results

The demographic data of the children is shown in **Table 1**. The children who received 6% sevoflurane were slightly older, taller, and heavier than the children of the 8% sevoflurane group, but the differences are not significant. 79% of the studied patients were male and 21% were female. There was no significant difference between the groups with regard to the ASA anaesthesia risk classes.

**Table 1:** Demographic data (mean ± standard deviation or frequencies).

	8% Sevoflurane	6% Sevoflurane	p
Age [years]	4.2±2.6	4.9±3.4	0.2413
Weight [kg]	17.3±6.5	20.7±12.2	0.0850
Height [cm]	102.8±17.5	108.1±23.2	0.2053
Female/Male [n]	9/41	12/38	0.6242
ASA [I/II/III]	34/15/1	34/14/2	1.000

Mask induction was well tolerated by all children. For airway management, in 44% of the children a tube and in 55% a laryngeal mask was used, one child was ventilated through a tracheostoma. No child had rhythmic, seizure-like movements.

The time from the start of the sevoflurane administration until LOC did not differ significantly between patients with 8% and 6% sevoflurane (42.2±17.5 s vs. 44.9 s ±14.0 s; p = 0.4073). An EEG stage of deep anaesthesia characterized by continuous delta waves smaller than 2.0 Hz appeared significantly earlier in the 8% group than in the 6% group (64.0±22.2 s vs. 77.9±20.0 s, p = 0.0022) (**Table 2**). The length of sections A and B was not statistically different with 6% and with 8% sevoflurane, although there was a tendency towards a shorter duration in the group with 8% sevoflurane compared to the group with 6% (**Table 2**).

**Table 2:** Time from the beginning of sevoflurane administration to loss of consciousness (LOC) and to continuous delta activity <2 Hz. Duration of sections A and B (mean ± standard deviation).

	8% Sevoflurane	6% Sevoflurane	p
LOC [s]	42.2±17.5	44.9±14.0	0.4073
Time from start of sevoflurane to start ofDelta <2 Hz [s]	64.0±22.2	77.9±20.0	0.0022
Duration section A [s]	393.2±154.7	448.2±122.7	0.0514
Duration section B [s]	607.5±309.2	669.7±351.3	0.3496

(Section A: start of sevoflurane administration until beginning of the propofol injection.

Section B: start of propofol injection until transition from a burst suppression or suppression pattern back to a continuous EEG.).

In section A, significantly more patients with 8% than with 6% sevoflurane had epileptiform potentials. 38 patients (76%) with 8% sevoflurane had epileptiform EEG patterns compared to 26 patients (52%) with 6% (p = 0.0106) (**Table 3**). When subgroups were built based on the number of seconds with epileptiform potentials (0 s, 1–2 s, 3–9 s, or >9 s with epileptiform potentials), a significant statistical difference was found between the two dosage groups (p = 0.0393) (**Table 4**).

**Table 3:** Number and percentage of patients without and with epileptiform activity in section A (start of sevoflurane until beginning of propofol bolus), (p = 0.0106).

Patients	8% Sevoflurane (n = 50)	6% Sevoflurane (n = 50)
Patients without epileptiform activity	12 (24%)	24 (48%)
Patients with epileptiform activity	38 (76%)	26 (52%)

**Table 4:** Number and percentage of patients who had 0, 1–2, 3–9 or >9 s with epileptiform activity in section A (start of sevoflurane until beginning of propofol bolus), (p = 0.0393).

Number of seconds with epileptiform activity	8% Sevoflurane (n = 50)	6% Sevoflurane (n = 50)
0 s	12 (24%)	24 (48%)
1–2 s	9 (18%)	6 (12%)
3–9 s	11 (22%)	7 (14%)
>9 s	18 (36%)	13 (26)%

The rhythmic EEG patterns PSR and/or PED were observed in 10 children (all had PSR) with 8% and in 7 children (4 with PSR, 3 with PSR and PED) with 6% sevoflurane in section A (**Table 5**).

**Table 5:** Number and percentage of patients with epileptiform EEG patterns in section A (start of sevoflurane until beginning of propofol bolus) and B (start of propofol bolus until end of burst suppression).

EEG patterns	8% Sevoflurane (n = 50)	6% Sevoflurane (n = 50)
**Section A**		
Delta with spikes	38 (76%)	26 (52%)
Rhythmic polyspikes (PSR)	10 (20%)	7 (14%)
Periodic epileptiform discharges (PED)	0 (0%)	3 (6%)
Suppression with spikes (SSP)	3 (6%)	3 (6%)
**Section B**		
Delta with spikes (DSP)	14 (28%)	14 (28%)
Rhythmic polyspikes (PSR)	2 (4%)	7 (14%)
Periodic epileptiform discharges (PED)	0 (0%)	1 (2%)
Suppression with spikes (SSP)	2 (4%)	2 (4%)

In section B, less children than in section A had epileptiform patterns, and no statistically significant difference was found with regard to the number of patients with and without epileptiform potentials in the groups with 8% and 6% sevoflurane (**Table 5**).

In section C, periods of more than 60 s with a high content of epileptiform activity occurred in 3 patients of the 8% sevoflurane group and 3 of the 6% group.

**Figure 2:**
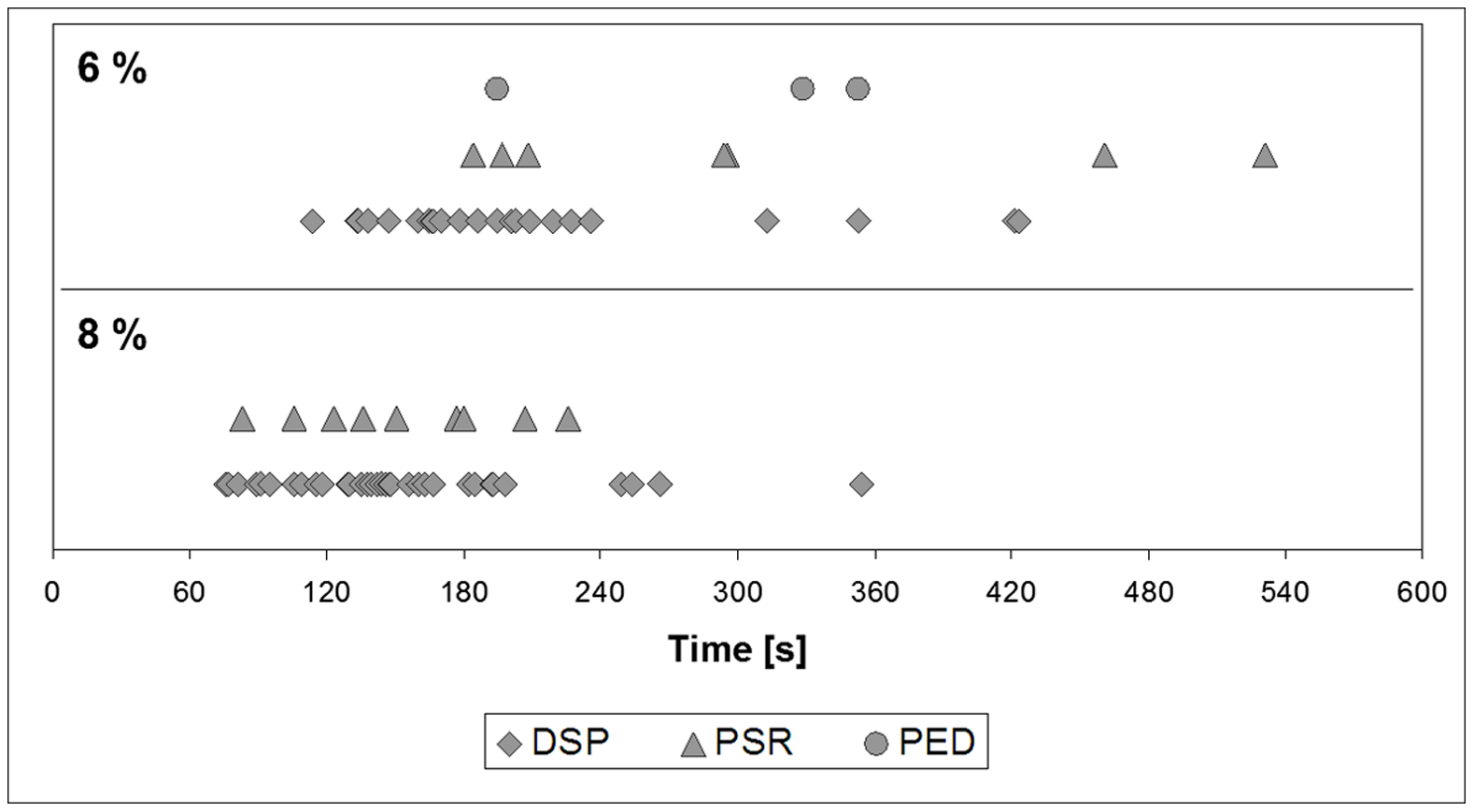
Time from start of sevoflurane administration to the first second with the patterns delta with spikes (DSP), rhythmic polyspikes (PSR), and periodic epileptiform discharges (PED) in the first 10 minutes of anaesthesia induction.

In section A, the median values for the time between the start of sevoflurane administration and the first second with the patterns DSP or PSR were smaller in patients with 8% compared to 6% sevoflurane. For the pattern PSR, the difference was statistically significant (p = 0.0051) (**Table 6** **,** **Figure 2** **,** **Table 7**). The times until PED and SSP started were not compared statistically between the groups, because the numbers of patients with these patterns were small.

**Table 6:** Time from start of sevoflurane to onset of epileptiform EEG patterns in section A (median [min – max]), (DSP: p = 0.1346; PSR: p = 0.0051).

Time to EEG pattern	8% Sevoflurane	6% Sevoflurane
Delta with spikes (DSP) [s]	146.5 [76–702]	182 [114–423]
Rhythmic polyspikes (PSR) [s]	163.5 [83–226]	294 [184–531]

**Table 7:** Time from start of sevoflurane to first epileptiform EEG pattern (median [min – max]), (p = 0.1393).

	8% Sevoflurane	6% Sevoflurane
Time to first epileptiformpattern [s]	144 [76–698]	182 [114–423]

**Figure 3:**
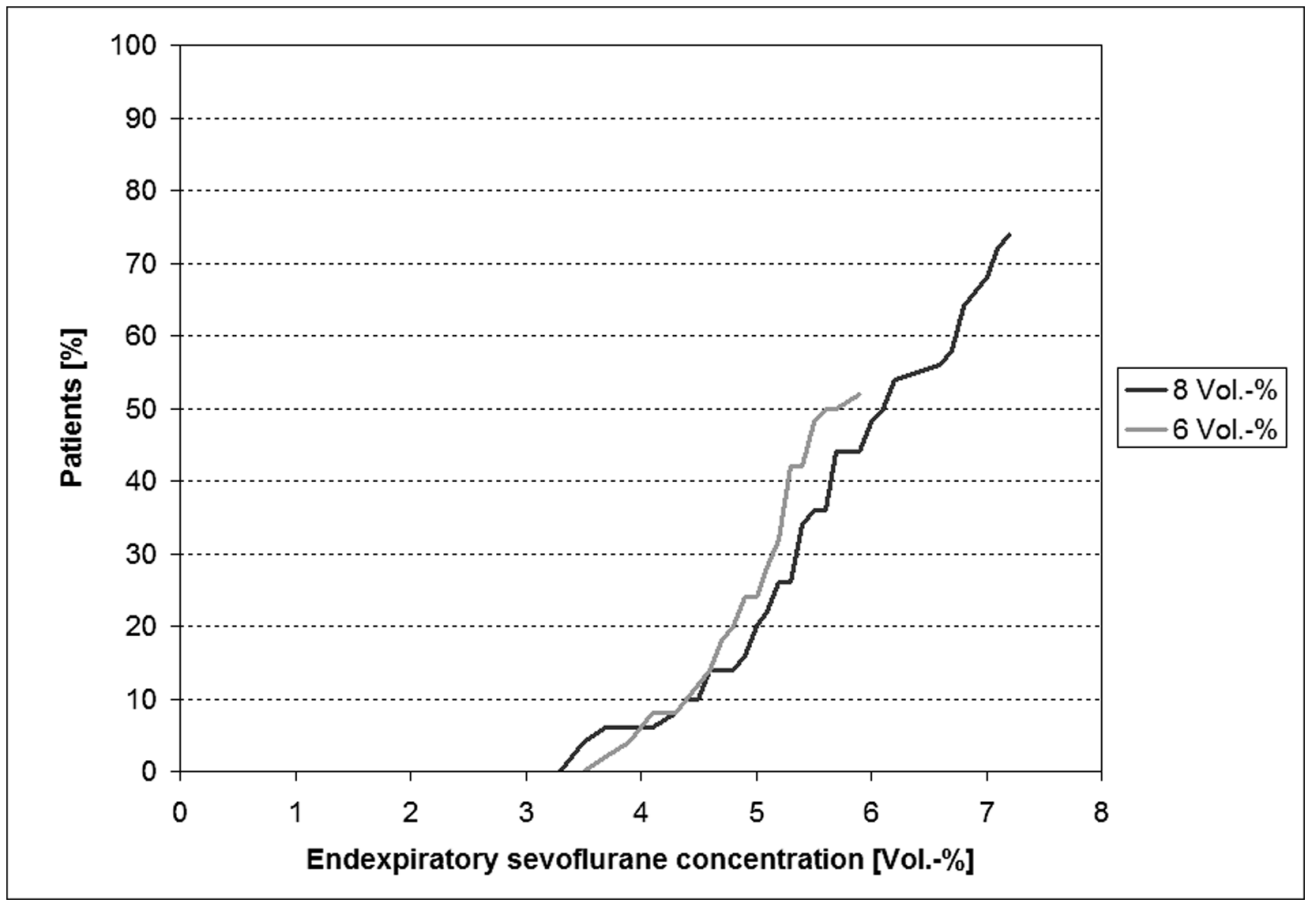
Endtidal sevoflurane concentrations before the first second with epileptiform activity and cumulative number of patients (%) with epileptiform potentials in section A (start of sevoflurane until start of propofol bolus).

The endexpiratory sevoflurane concentration was noted at 1-minute intervals for 5 minutes after the start of the sevoflurane administration. It was analysed which concentration was reached before the first epileptiform pattern appeared. Up to an endtidal concentration of 6% sevoflurane, the number of children with epileptiform potentials was similar in the groups with 6% and with 8% sevoflurane (p = 0.3708) **(** **Figure 3** **)**.

For each child without epileptiform potentials, the highest endtidal sevoflurane concentration in the 5-minute period after the start of the sevoflurane administration was determined. In children with 6 and 8%, these values were at least 4.9 and 5.7%, resp., i. e., the children without epileptiform EEG activity reached endexpiratory sevoflurane concentrations that were in the range of the concentrations measured in children with epileptiform potentials.

Apart from epileptiform patterns, a pattern consisting of rhythmic, high amplitude delta waves <2 Hz could be observed in some of the EEGs. With 6% compared to 8% sevoflurane, the number of patients with this pattern (21 vs. 27), the time from start of sevoflurane until the beginning of this pattern (104.1±73.8 vs. 108.3±41.8 s) and the number of seconds with this pattern (5.2±12.5 vs. 6.8±10.1 s) was not statistically different.

## Discussion

The study showed that induction of anaesthesia with 6% sevoflurane in children was accompanied by less epileptiform activity compared to administration of 8% sevoflurane: less children had epileptiform activity, and epileptiform potentials tended to appear later in the course of the induction with 6% sevoflurane.

Except for oral premedication with midazolam, no other drug was used in the beginning of the induction. Therefore, the fact that epileptiform EEG potentials occurred can be attributed to the use of sevoflurane.

The benzodiazepine midazolam was used for premedication in other studies on the EEG effects of sevoflurane. Benzodiazepines have anticonvulsant properties [16]. Epileptiform patterns were observed in the studies on sevoflurane EEG effects by Vakkuri et al. [4] and Sonkajärvi et al. [7] in spite of premedication with midazolam. All children in the own investigation received midazolam 0.5 mg/kg (maximum dose 10 mg) as oral premedication.

Several authors examined the influence of inhaled and endtidal concentrations of sevoflurane on the occurrence of epileptiform EEG patterns in adults and in children. No seizure-like changes in the EEG were found by Nieminen et al. [17] in children during maintenance of anaesthesia with an endtidal concentration of 2% sevoflurane. Julliac et al. [2] gave 8% sevoflurane for induction of anaesthesia in 40 adult patients. The endtidal sevoflurane concentration was maintained at 4% or 2%, and in the patients with 4% endtidal sevoflurane different ventilation modes were used. In each of the three groups with a maintenance concentration of 4% endtidal sevoflurane, 3 to 5 of 10 patients had epileptiform potentials, whereas in the group with 2% only 1 of 10 patients had such EEG activity. The difference was not statistically significant. Jääskeläinen et al. [18] demonstrated in non-epileptic adult volunteers who received the three endtidal concentrations 2%, 3% and 4% sevoflurane for 30 min each that sevoflurane produced epileptiform discharges in a dose-dependent manner. At 2% endtidal sevoflurane, epileptiform discharges occurred in 7 of 8 persons. At 3%, they occurred in all persons, additionally, 1 person had repetitive epileptiform discharges. At 4% endtidal sevoflurane, all persons had epileptiform discharges and repetitive epileptiform discharges, 3 had electrographic seizures, in 1 case accompanied with clinical motor seizures. It should be mentioned that the adult participants in the studies by Julliac et al. [2] and Jääskeläinen et al. [18] were not premedicated and that they did not receive N_2_O or opioids during the study periods.

In children receiving sevoflurane in 100% O_2_ under steady-state conditions, the lowest endtidal concentration causing major epileptiform signs, which were characterized by rhythmicity, was 4.3% in a study by Gibert et al. [14]. The concentration was 4.5% when sevoflurane was given together with a mixture of N_2_O/O_2_ (50∶50) or when 20 µg/kg alfentanil had been given before the study period [14].

In the own analysis, the lowest endtidal sevoflurane concentrations before the occurrence of epileptiform patterns during the induction were between 3.4 and 4.0%. The concentrations reported by Gibert et al. [14] are higher. Reasons for the difference may be that in the own analysis the endtidal concentrations were already noted shortly before the epileptiform patterns appeared, and that the first epileptiform patterns, not only major epileptiform patterns, were included.

The cases of patients from the own analysis who developed episodes of more than 60 s length with a higher content of epileptiform activity in section C illustrate that rhythmic patterns (PSR, PED) tend to occur under higher sevoflurane concentrations than the pattern DSP. Two patients had such episodes after the termination of EEG suppression at the beginning of section C. Both had DSP and the endtidal sevoflurane concentration was near 3.0% in one child and 3.5 to 3.6% in the other child. During the course of section C, DSP and few PSR occurred in a 5-year-old child with 3.4–3.3% sevoflurane, a 1-year-old child had DSP and SSP after the endtidal sevoflurane had been 4.4% and decreased from about 4.2 to 3.5%. In a 6-year-old patient DSP and PSR occurred when the endtidal sevoflurane concentration was increased from 4.3 to 4.6%. DSP, PSR, and some PED, occurred in a 4-year-old child under increasing endtidal sevoflurane concentrations between 3.8 and 4.3%.

Hyperventilation is discussed in the literature as one factor that may activate electroencephalogram abnormalities during sevoflurane administration [4]. The children in the own analysis were normoventilated after the LOC. Epstein et al. [19] used 8% sevoflurane in a 2∶1 nitrous oxide:oxygen mixture (N_2_O:O_2_) for induction of anaesthesia and reported that in uncooperative children the time from start of induction to loss of the eyelid reflex was shorter than in cooperative children. The authors assumed that this resulted from a higher minute ventilation in uncooperative relative to cooperative patients. In the own data set, the time from start of induction to the occurrence of slow EEG waves, which characterize a deep EEG stage of anaesthesia, was shortest in the most agitated children (0.7±0.3 min), was longer in only moderately crying children (1.1±0.3 min), and was longest in children who were calm (1.3±0.4 min), but the portion of children with epileptiform EEG activity was not significantly different in the three groups (p = 0.8775).

Julliac et al. [2] reported that women had a higher risk to develop epileptiform activity than men, but in the own data set no gender effect was found. In another study from the own department on EEG effects during induction of anaesthesia with sevoflurane in children, also no gender effect was found [13].

The own analysis indicates a clear relationship between the endtidal sevoflurane concentration and the presence of epileptiform activity. **Figure 3** shows that with increasing sevoflurane concentrations the numbers of children with epileptiform activity became greater. Up to a concentration of 6%, the cumulative percentages of children with 6% and 8% sevoflurane who had epileptiform potentials were similar (p = 0.3708). In total, in our analysis, more children with 8% sevoflurane compared to 6% had epileptiform potentials in their EEGs, because higher endtidal sevoflurane concentrations were reached with 8% sevoflurane.

With 6% sevoflurane, the longest time from start of sevoflurane until an EEG pattern of deep anaesthesia, which was characterized by delta waves <2 Hz, appeared was 126 s, and the longest time from start of sevoflurane to LOC was 79 s. Accordingly, reducing the administration time of 6% sevoflurane e. g. to 3 min, as with 8% sevoflurane, would have been safe with regard to depth of hypnosis. Shortening the administration time of 6% sevoflurane could probably have reduced the number of children with epileptiform potentials at least to some extent. In 14 of the 26 children of the 6% sevoflurane group with epileptiform potentials the first second with epileptiform potentials occurred later than 3 min after the start of the sevoflurane administration.

Schultz et al. [13] reported on the incidence of epileptiform EEG activity related to brief administration of 8% sevoflurane in 100% O_2_ in children. Immediately after the LOC the inhaled concentration was reduced to 4%. Epileptiform EEG patterns were detected in 20% of the patients. The administration time of 8% sevoflurane was 73±26 s on average.

Constant et al. [5] and Gibert et al. [14] recommend to use sevoflurane with a maximum of 1.5 MAC for maintenance of anaesthesia to reduce the possibility that major epileptiform signs, which are characterized by rhythmicity, occur in the EEG. The authors argue that it is reasonable to avoid circumstances favouring the emergence of these signs, particularly, because there are concerns about the safety of anaesthetic agents in the brain of young children.

The MAC value of sevoflurane given without N_2_O is reported to be 2.8% in children from 6 months to below 3 years, 2.5% between 3 and 12 years and 2.6% at an age of 25 years [20]. The corresponding 1.5 MAC values are 4.2%, 3.75%, and 3.9%.

In patients with a history of epilepsy, epileptiform EEG activity may be observed even with lower sevoflurane concentrations. Iijima et al. [21] showed that during administration of sevoflurane from 1 to 2 MAC, adult patients with a history of epileptic disorders had an increasing amount of epileptiform activity in their EEGs. Under the same conditions, no spike activity was observed in patients without epilepsy.

In normoventilated persons without epileptic disorders, lower endtidal concentrations of sevoflurane indicate a lower risk for epileptiform potentials than higher concentrations [2], [14], [17], [18]. But, although the risk for epileptiform activity decreases when the endtidal sevoflurane concentration is kept relatively low, the individual reaction cannot be foreseen. Voss et al. [16] gave the recommendation to consider EEG monitoring when sevoflurane is used.

### Conclusions

In the own analysis, using 6% sevoflurane for induction of anaesthesia, and thus limiting the maximum achievable endtidal concentration of sevoflurane, resulted in a lower number of children with epileptiform EEG patterns compared to inductions of anaesthesia with 8% sevoflurane. EEG monitoring can be used to observe individual reactions to sevoflurane [16], [20].

The probability of developing epileptiform activity rose with increasing endtidal sevoflurane concentrations, and up to an endtidal concentration of 6% the cumulative numbers of children with epileptiform potentials were similar with 6 and 8% sevoflurane. There was no significant difference in the time to the clinically observable loss of consciousness, between 8% and 6% sevoflurane, the mean time to an EEG stage of deep anaesthesia was about 14 s longer with 6% compared to 8% sevoflurane (77.9±20.0 s vs. 64.0±22.2 s).

The own analysis and data from the literature imply that avoiding higher endtidal sevoflurane concentrations can reduce the risk for the occurrence of epileptiform activity during induction of anaesthesia in children. This may be achieved by using inhaled sevoflurane concentrations below 8 Vol.-% and limiting administration times.
